# Associations of dietary factors with gastric cancer risk: insights from NHANES 2003–2016 and mendelian randomization analyses

**DOI:** 10.3389/fgene.2024.1377434

**Published:** 2024-05-02

**Authors:** Yigang Zhang, Sen Wang, Qingya Li, Hongda Liu, Zhe Xuan, Fengyuan Li, Zheng Li, Yiwen Xia, Tianlu Jiang, Penghui Xu, Lang Fang, Linjun Wang, Diancai Zhang, Hao Xu, Li Yang, Zekuan Xu

**Affiliations:** ^1^ Department of General Surgery, The First Affiliated Hospital of Nanjing Medical University, Nanjing, China; ^2^ Collaborative Innovation Center for Cancer Personalized Medicine, Nanjing Medical University, Nanjing, China; ^3^ The Institute of Gastric Cancer, Nanjing Medical University, Nanjing, China

**Keywords:** gastric cancer, dietary factors, NHANES, MUFA, Mendelian randomization

## Abstract

**Background:** Gastric cancer (GC) continues to be one of the leading causes of cancer-related deaths globally. Diet significantly influences the incidence and progression of GC. However, the relationship between dietary intake and GC is inconsistent.

**Methods:** A study was conducted with adults who participated in the National Health and Nutrition Examination Survey (NHANES) from 2003 to 2016 to investigate possible associations between 32 dietary factors and GC. To further detect potential causal relationships between these dietary factors and the risk of GC, a two-sample Mendelian randomization (MR) analysis was conducted. The primary method employed was the inverse variance weighted (IVW) analysis, and its results were further validated by four other methods.

**Results:** Of the 35,098 participants surveyed, 20 had a history of GC. Based on the results of weighted logistic multivariate analysis, it was observed that there was a positive correlation between total fat intake [odds ratio (OR) = 1.09, 95% confidence interval (CI): (1.01–1.17), *p* = 0.03] and GC as well as negative association of dietary monounsaturated fatty acids (MUFAs) intake [OR = 0.83, 95% CI: (0.76–0.92), *p* < 0.001]. Further evaluations of the odds of GC across the quartiles of dietary MUFAs showed that the top quartile of total MUFA intake was associated with a lower likelihood of GC in three different models [model1: OR = 0.03, 95% CI: (0.00–0.25), *p* < 0.01; model2: OR = 0.04, 95% CI: (0.00–0.38), *p* = 0.01; model3: OR = 0.04, 95% CI: (0.00–0.40), *p* = 0.01]. For the MR analyses, genetic instruments were selected from the IEU Open GWAS project; IVW analysis showed that GC risk was not associated with MUFAs [OR = 0.82, 95% CI: (0.59–1.14), *p* = 0.23] or the ratio of MUFAs to total fatty acids [OR = 1.00, 95% CI: (0.75–1.35), *p* = 0.98]. Similar results were observed when using the other MR methods.

**Conclusion: **The NHANES study revealed that consuming MUFAs was linked to a lower risk of GC, although the results of MR analyses do not provide evidence of a causal relationship. Additional research is therefore necessary to clarify these findings.

## Introduction

The rapidly growing global incidence of gastric cancer (GC) presents a significant public health challenge as it remains one of the leading cause of cancer-related mortality (Siegel et al., 2023). Despite advancements in early screening and therapeutic approaches, patients with advanced GC still have poor prognosis (Thrift et al., 2023). The development of GC is multifactorial and involves influences from factors, such as diet, environment, and genetics, with the dietary factors being of particular significance (Bouras et al., 2022). Based on reflection of an old Chinese proverb that “illness comes from the mouth,” it is imperative to look into the associations between dietary factors and GC. By gaining a deeper understanding of their relationship, efforts can be made to modify dietary patterns to potentially reduce the incidence of GC.

Recent studies have identified several dietary factors that may be associated with GC; of these, high glucose levels in the body are believed to be linked to greater incidence of malignancies, including GC (Tay et al., 2021). Similarly, increased fat intake has been identified as another important dietary habit that is carcinogenic and potentially related to GC (Kyrgiou et al., 2017). Protein is a fundamental component necessary for body composition and is regarded as a pivotal nutrient for GC patients (Ouyang et al., 2018; Kubota et al., 2020). Furthermore, multiple studies have highlighted the strong positive association between high salt consumption and GC, particularly with respect to salt-preserved foods (Kurosawa et al., 2006; D'Elia et al., 2012). For instance, a recent study reported that high intake of salted fish was linked to an elevated risk of GC (Bouras et al., 2022). On the other hand, high consumption of vitamin C, carotenoids, and other antioxidants, which have the potential to mitigate oxidative damage, has been reported to confer protective effects against the incidence of GC (Kong et al., 2014; Kim et al., 2018; Chen et al., 2021). However, a recent clinical trial found no significant interactions between vitamin supplements and GC incidence (Guo et al., 2020). Nevertheless, it is important to note that most current studies concentrate on a single dietary factor while neglecting the complexity, diversity, and interactions of different dietary intakes. As a result, these reports on the associations between dietary factors and GC may be one-sided. Therefore, it is imperative to shift the focus toward examining food groups or dietary patterns by taking into account multiple dietary factors and conducting comprehensive studies to gain a more holistic understanding of the associations between diet and GC.

Mendelian randomization (MR) is an approach that utilizes genetic variants as instrumental variables (IVs) and offers several advantages over observational studies; it has the potential to circumvent residual confounding and reverse causality, thereby providing a more reliable approach for evaluating causal relationships (Boyko, 2013; Sekula et al., 2016). Therefore, MR was employed in this study to further investigate the causal relationships between some dietary factors of interest and the risk of GC.

Thus, this study aims to investigate the links between dietary factors and the risk of GC by integrating an observational study and two-sample MR analyses. The main goal of this work was to establish a theoretical basis for the prevention and treatment of GC through the improvement of dietary habits.

## Methods

### Study design and population in NHANES

The data for this cross-sectional study were extracted from the National Health and Nutrition Examination Survey (NHANES), a multistage stratified composite design survey on the health and nutritional information of a representative selection of the non-institutionalized U.S. population, conducted by the National Centers for Health Statistics (NCHS) of the Centers for Disease Control and Prevention (CDC). The observations from seven consecutive NHANES surveys (2003–2004, 2005–2006, 2007–2008, 2009–2010, 2011–2012, 2013–2014, and 2015–2016) were combined into a single analytic sample; thus, a total of 35,098 eligible participants above the age of 18 years, who were interviewed regarding their medical conditions and dietary intakes, were included in this study. The participants who had incomplete information were excluded (n = 6,809).

### Variable selection in NHANES

The diagnoses of GC were defined using two items on the Medical Status Questionnaire: “Have you ever been told by a doctor or other health professional that you had cancer or malignancy?” and “What kind of cancer was it?” Answers that indicated only “stomach cancer” were classified as the outcome variables. Some demographic covariates, including age, sex, race, education, smoking status, weight, and body mass index (BMI), were also assessed.

The study participants were asked by trained interviewers to recall two consecutive 24-h dietary periods (day 1 and day 2) to assess the total dietary intakes through comprehensive reference to the NHANES. The present study only included dietary recalls for day 1 as those for day 2 had more missing values. A total of 32 dietary factors from the dietary questionnaire in the NHANES database were included in this study as follows: energy (kcal), protein (g), carbohydrate (g), total sugars (g), dietary fibers (g), total fat (g), saturated fatty acids (SFAs, g), monounsaturated fatty acids (MUFAs, g), polyunsaturated fatty acids (PUFAs, g), cholesterol (mg), vitamin A (µg), retinol (µg), alpha-carotene (µg), beta-carotene (µg), vitamin B1 (thiamin, mg), vitamin B2 (riboflavin, mg), vitamin B3 (niacin, mg), vitamin B6 (mg), folate (µg), vitamin B12 (µg), vitamin C (µg), vitamin E (mg), vitamin K (µg), calcium (mg), phosphorus (mg), magnesium (mg), iron (mg), zinc (mg), copper (mg), sodium (mg), potassium (mg), and selenium (µg).

### Data sources for genetic instruments

The genome-wide association study (GWAS) data analyzed in the present study was obtained from the IEU open GWAS project supported by the MRC Integrative Epidemiology Unit (IEU) at the University of Bristol, collated and analyzed GWAS data from the UK Biobank, FinnGen biobank, and published articles. The single-nucleotide polymorphisms (SNPs) at the genome‐wide significance level (*p* < 5 × 10^−8^) used in this study included MUFAs (GWAS ID: met-d-MUFA, sample size: 114,999, number of SNPs: 12,321,875, population: European, gender: both) and ratio of MUFAs to total fatty acids (GWAS ID: met-d-MUFA_pct, sample size: 114,999, number of SNPs: 12,321,875, population: European, gender: both). The data on SNPs associated with GC (GWAS ID: finn-b-C3_STOMACH, sample size: 218,792, samples with GC: 633, number of SNPs: 16,380,466, population: European, gender: both) were also extracted from the IEU open GWAS project (https://gwas.mrcieu.ac.uk/).

### Genetic instrument selection

The SNPs in linkage disequilibrium (defined as *r* ^2^ > 0.001 or clump distance <10,000 kb) and those having weaker associations with exposure were excluded, leaving 66 independent SNPs as the IVs for MUFAs and 66 for the ratio of MUFAs to total fatty acids. The F-statistic was used to ensure strong association between the SNPs and exposure. The detailed information on the selected SNPs is presented in Supplementary Tables S1,S2.

### Statistical analysis

The data in the current study were obtained and statistically evaluated using R 4.1.1 (R Foundation, Vienna, Austria). The NHANES study population was divided into two groups in accordance with the presence or absence of a history of GC, and characteristics were determined for comparison between the groups. Continuous variables were expressed in terms of the median and interquartile range (IQR) as they did not obey a normal distribution. Significance differences between the two groups were evaluated using the Wilcoxon rank-sum test. Frequency and percent were used to describe the categorical variables, and the distribution of the categorical variables was appropriately compared using the Pearson chi-squared test.

Considering the stratified multistage probabilistic sampling approach of the NHANES, the “survey” package was used to adjust the complex sampling weights in the analyses. The two-year cycle weights were divided by seven to reflect the 14 survey years. Weighted logistic multivariate analysis was used to explore the associations between the dietary factors and GC. Three different models were used to decrease the influences of the confounders, where the first model was the crude model; the second model was adjusted for age, sex, and race, and the third model was adjusted for age, sex, race, education, smoking status, and BMI. The odds ratio (OR) and 95% confidence interval (CI) were used to assess the associations.

For the MR analyses, the “TwoSampleMR” package was used to conduct the inverse variance weighted (IVW) analysis as the primary method of assessing the causal effect between MUFAs and GC risk. The IVW model is considered to have the strongest ability to detect causation in the two-sample MR analysis (Hartwig et al., 2017). MR–Egger, weighted-median, simple mode, and weighted mode were also implemented to validate the results from the IVW analysis. The possible heterogeneity and directional pleiotropy were assessed through the Cochrane Q test and intercept from MR–Egger (Qian et al., 2020). The leave-one-out sensitivity analysis was also conducted, and a *p*-value <0.05 (two-sided) was considered to be statistically significant in this study.

## Results

### Characteristics of included participants

**FIGURE 1:**
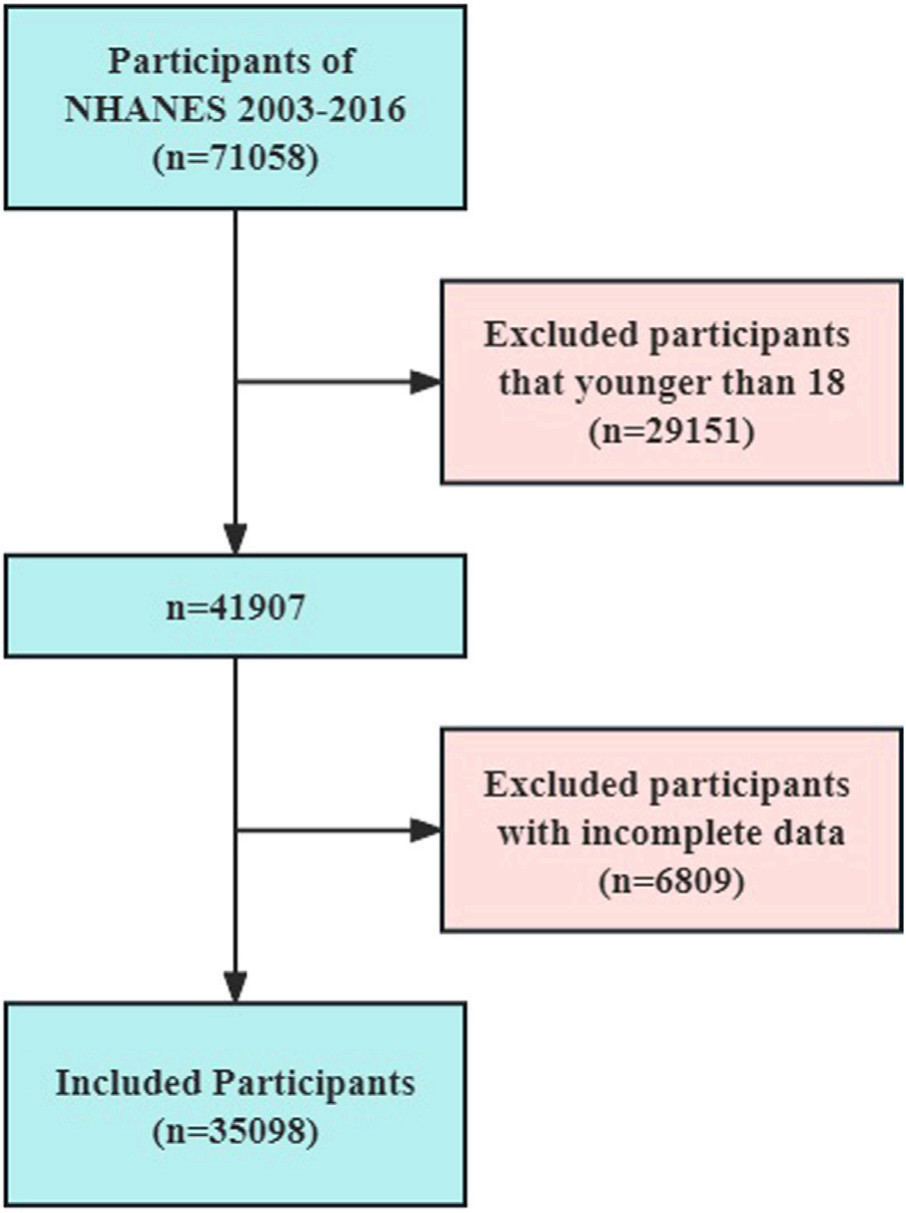
Flowchart of the NHANES study participants.

A total of 35,098 individuals (weighted n = 219,465,579) over 18 years of age were selected for this study through the NHANES database. The flowchart illustrating the selection process of the participants is depicted in Figure 1. Among these participants, 20 people representing 105,634 individuals reported having a history of GC. The characteristics of these individuals were then stratified based on the presence or absence of GC, as shown in Table 1. The analysis revealed that individuals with GC were older (53 vs 46 years, *p* < 0.0001), had lower weights (67.40 vs 79.30 kg, *p* = 0.02), and had lower educational attainment (*p* = 0.03) compared to those without GC.

**TABLE 1:** General characteristics of the adults included in this study stratified by the presence or absence of a history of GC.

Variable	Total (unweighted n = 35,098, weighted n = 219,465,579)	GC (unweighted n = 20, weighted n = 105,634)	No GC (unweighted n = 35,078, weighted n = 219,359,945)	*p*-value
Age (median (IQR))	46 (32.59)	53 (48.67)	46 (32.59)	**< 0.0001**
Sex (%)				0.06
Male	48.16	22.06	48.17	
Female	51.84	77.94	51.83	
Race (%)				0.51
Non-Hispanic Black	11.34	20.95	11.34	
Non-Hispanic White	68.44	71.97	68.44	
Mexican American	8.48	3.53	8.49	
Other Hispanic	4.9	0	4.9	
Other race	6.84	3.56	6.84	
Education (%)				**0.03**
Less than high school	16.97	27.5	16.96	
High school graduate	23.64	6.51	23.65	
Some college	31.6	58.87	31.59	
College graduate or above	27.79	7.12	27.8	
Smoking status (%)				0.9
No	78.36	76.9	78.36	
Yes	21.64	23.1	21.64	
Weight (median (IQR))	79.20 (67.00.93.70)	67.40 (67.40.81.40)	79.30 (67.00.93.70)	**0.02**
BMI (median (IQR))	27.61 (24.03.32.20)	25.90 (24.40.28.20)	27.61 (24.02.32.20)	0.1

Data are expressed as median (IQR) for skewed variables and percentage (%) for categorical variables. *p*-value for skewed variables was assessed by the Wilcoxon rank-sum test, and *p*-value for the categorical variables was determined using the Pearson chi-squared test.

The bold values mean *p* < 0.05.

### Dietary intakes and risk of GC

Table 2 represents the dietary intakes of the participants with and without GC. Individuals with GC consumed less energy (*p* < 0.001), carbohydrates (*p* = 0.01), dietary fibers (*p* < 0.0001), total fats (*p* = 0.03), MUFAs (*p* < 0.01), PUFAs (*p* = 0.04), vitamin B1 (*p* < 0.0001), vitamin B3 (*p* < 0.0001), vitamin B6 (*p* < 0.0001), folate (*p* < 0.0001), vitamin E (*p* < 0.0001), vitamin K (*p* < 0.0001), phosphorus (*p* = 0.01), magnesium (<0.0001), iron (*p* < 0.0001), zinc (*p* < 0.01), copper (*p* = 0.01), sodium (*p* = 0.02), and selenium (*p* = 0.03).

**TABLE 2:** Comparison of dietary intakes of persons with and without self-reported GC.

Variable	Total	GC	No GC	*p*-value
Energy (kcal)	1,988.00 (1,479.00–2,649.00)	1,615.00 (1,038.00–1615.00)	1,989.00 (1,479.00–2,649.00)	**< 0.001**
Proteins (g)	75.81 (54.17–103.46)	51.95 (44.83–97.60)	75.82 (54.19–103.48)	0.15
Carbohydrates (g)	236.50 (171.86–319.67)	192.76 (94.95,218.33)	236.56 (171.88–319.74)	**0.01**
Total sugars (g)	99.26 (61.94–150.26)	111.15 (38.91,111.15)	99.26 (61.98–150.26)	0.41
Dietary fibers (g)	14.70 (9.70–21.30)	9.10 (5.60–9.30)	14.70 (9.70–21.30)	**< 0.0001**
Total fats (g)	74.42 (50.20–105.17)	56.02 (29.94–71.58)	74.44 (50.20–105.17)	**0.03**
SFAs (g)	23.69 (15.21–35.20)	17.39 (15.33–26.14)	23.69 (15.21–35.20)	0.19
MUFAs (g)	26.49 (17.38–38.30)	21.63 (9.29–22.84)	26.50 (17.38–38.30)	**0.002**
PUFAs (g)	15.82 (10.02–23.87)	10.90 (2.47–15.98)	15.82 (10.02–23.87)	**0.04**
Cholesterol (mg)	223.00 (131.00–380.00)	272.00 (185.00–556.00)	223.00 (131.00–380.00)	0.47
Vitamin A (µg)	494.00 (274.00–812.00)	464.00 (464.00–857.00)	494.00 (274.00–812.00)	0.43
Retinol (µg)	334.00 (171.00–566.00)	443.00 (321.00–453.00)	334.00 (171.00–566.00)	0.08
Alpha carotene (µg)	47.00 (10.00–244.00)	13.00 (0.00–158.00)	47.00 (11.00–244.00)	0.48
Beta carotene (µg)	779.00 (300.00–2,312.00)	555.00 (214.00–4,768.00)	779.00 (300.00–2,312.00)	0.78
Vitamin B1 (mg)	1.46 (1.02–2.03)	0.95 (0.63–1.13)	1.46 (1.02–2.03)	**< 0.0001**
Vitamin B2 (mg)	1.97 (1.36–2.73)	2.04 (1.88–2.06)	1.97 (1.36–2.73)	0.51
Vitamin B3 (mg)	22.73 (15.89–31.80)	13.24 (13.24–18.81)	22.74 (15.90–31.81)	**< 0.0001**
Vitamin B6 (mg)	1.77 (1.18–2.54)	0.91 (0.65–1.45)	1.77 (1.18–2.54)	**< 0.0001**
Folate (µg)	354.00 (240.00–511.00)	247.00 (170.00–287.00)	354.00 (240.00–512.00)	**< 0.0001**
Vitamin B12 (µg)	3.96 (2.25–6.47)	2.79 (2.77–4.28)	3.96 (2.25–6.47)	0.15
Vitamin C (mg)	53.40 (21.90–115.60)	26.90 (9.30–110.50)	53.50 (21.90–115.60)	0.38
Vitamin E (mg)	6.72 (4.30–10.27)	4.18 (1.81–4.69)	6.72 (4.31–10.27)	**< 0.0001**
Vitamin K (µg)	63.80 (35.80–119.30)	34.10 (25.00–49.30)	63.80 (35.80–119.40)	**< 0.0001**
Ca (mg)	834.00 (546.00–1,223.00)	810.00 (571.00–1,009.00)	834.00 (546.00–1,223.00)	0.55
P (mg)	1,263.00 (915.00–1,717.00)	966.00 (943.00–1,286.00)	1,263.00 (915.00–1,717.00)	**0.01**
Mg (mg)	275.00 (198.00–370.00)	179.00 (179.00–264.00)	275.00 (198.00–370.00)	**< 0.0001**
Fe (mg)	13.27 (9.33–18.92)	8.80 (5.47–10.69)	13.28 (9.33–18.92)	**< 0.0001**
Zn (mg)	10.17 (6.97–14.71)	6.47 (5.14–9.87)	10.17 (6.98–14.71)	**0.002**
Cu (mg)	1.14 (0.82–1.58)	0.55 (0.54–1.00)	1.14 (0.82–1.58)	**0.01**
Na (mg)	3,217.00 (2,287.00–4,406.00)	2,856.00 (1,902.00–3,101.00)	3,218.00 (2,287.00–4,408.00)	**0.02**
K (mg)	2,535.00 (1,820.00–3,365.00)	1,492.00 (1,350.00–2,531.00)	2,536.00 (1,821.00–3,365.00)	0.06
Se (µg)	101.90 (70.40–142.10)	80.40 (63.30–103.80)	101.90 (70.40–142.20)	**0.03**

*p*-value was determined by the Wilcoxon rank-sum test, median (IQR).

The bold values mean *p* < 0.05.

The correlations between the aforementioned dietary intakes and risk of GC via logistic regression analysis after adjustment for multiple potential confounders are described in Table 3. The dietary total fat intake [OR = 1.09, 95% CI: (1.01–1.17), *p* = 0.03] was positively associated with GC; meanwhile, dietary MUFA intake [OR = 0.83, 95% CI: (0.76–0.92), *p* < 0.001] was negatively associated with GC. After adjusting for age, sex, and race (model2) as well as age, sex, race, education, smoking status, and BMI (model3), the dietary total fat intake [model2: OR = 1.08, 95% CI: (1.01–1.16), *p* = 0.02; model3: OR = 1.08, 95% CI: (1.01–1.16), *p* = 0.03] was still associated with higher odds of GC, and the dietary MUFA intake [model2: OR = 0.83, 95% CI: (0.75–0.91), *p* < 0.001; model3: OR = 0.83, 95% CI: (0.76–0.92), *p* < 0.001] was still associated with a lower risk of GC. The average total fat intake of the participants with GC in this study was still within the 25%–35% range recommended by the 2020–2025 Dietary Guidelines for Americans (Phillips, 2021). Despite the positive association between total fat intake and GC, it is important to note that the lower daily energy intakes of those with GC than without GC may have contributed to this relationship. Therefore, it is plausible that this association may be influenced by the differences in daily energy intakes between the two groups.

**TABLE 3:** Associations between dietary intakes and GC.

Variable	Model1		Model2		Model3	
	OR (95% CI)	*p*-value	OR (95% CI)	*p*-value	OR (95% CI)	*p*-value
Energy (kcal)	0.9972 (0.9918–1.0027)	0.3180	0.9978 (0.9928–1.0027)	0.3724	0.9976 (0.9922–1.0031)	0.3838
Carbohydrates (g)	1.0073 (0.9834–1.0318)	0.5471	1.0072 (0.9850–1.0299)	0.5227	1.0071 (0.9831–1.0317)	0.5596
Dietary fibers (g)	0.9574 (0.8970–1.0220)	0.1885	0.9495 (0.8813–1.0230)	0.1708	0.9605 (0.8836–1.0441)	0.3393
Total fats (g)	1.0866 (1.0099–1.1691)	**0.0266**	1.0818 (1.0114–1.1572)	**0.0226**	1.0820 (1.0069–1.1627)	**0.0321**
MUFAs (g)	0.8343 (0.7572–0.9192)	**<0.001**	0.8302 (0.7546–0.9135)	**<0.001**	0.8338 (0.7562–0.9194)	**<0.001**
PUFAs (g)	0.9688 (0.8677–1.0817)	0.5695	0.9701 (0.8634–1.0898)	0.6049	0.9727 (0.8636–1.0955)	0.6439
Vitamin B1 (mg)	0.8809 (0.1363–5.6913)	0.8929	0.8134 (0.1107–5.9757)	0.8373	0.8181 (0.1002–6.6778)	0.8495
Vitamin B3 (mg)	0.9955 (0.8870–1.1172)	0.9378	1.0059 (0.9016–1.1222)	0.9151	1.0069 (0.9113–1.1125)	0.8914
Vitamin B6 (mg)	0.6444 (0.3578–1.1605)	0.1413	0.6016 (0.3416–1.0592)	0.0776	0.5989 (0.3137–1.1432)	0.1185
Folate (µg)	0.9995 (0.9957–1.0034)	0.8137	1.0000 (0.9962–1.0037)	0.9899	0.9999 (0.9957–1.0041)	0.9707
Vitamin E (mg)	0.9831 (0.8378–1.1535)	0.8325	0.9804 (0.8212–1.1705)	0.8249	0.9847 (0.8317–1.1659)	0.8565
Vitamin K (µg)	0.9927 (0.9790–1.0065)	0.2951	0.9916 (0.9754–1.0080)	0.3073	0.9917 (0.9765–1.0072)	0.2897
P (mg)	1.0010 (0.9982–1.0038)	0.4776	1.0011 (0.9984–1.0037)	0.4293	1.0011 (0.9984–1.0039)	0.4174
Mg (mg)	1.0026 (0.9943–1.0109)	0.5358	1.0023 (0.9947–1.0100)	0.5479	1.0018 (0.9948–1.0089)	0.6067
Fe (mg)	0.9477 (0.8548–1.0507)	0.3037	0.9304 (0.8349–1.0367)	0.1885	0.9317 (0.8549–1.0153)	0.1053
Zn (mg)	1.0073 (0.9242–1.0979)	0.8666	1.0141 (0.9226–1.1146)	0.7695	1.0142 (0.9312–1.1047)	0.743
Cu (mg)	0.8428 (0.0795–8.9384)	0.8859	0.7847 (0.0777–7.9216)	0.8353	0.7996 (0.1129–5.6627)	0.8207
Na (mg)	1.0002 (0.9995–1.0008)	0.5906	1.0003 (0.9996–1.0009)	0.4116	1.0003 (0.9997–1.0009)	0.3561
Se (µg)	1.0046 (0.9786–1.0313)	0.7304	1.0043 (0.9785–1.0308)	0.7433	1.0042 (0.9792–1.0298)	0.7414

OR, odds ratio; CI, confidence interval.

Model1: crude model.

Model2: adjusted for age, sex, and race.

Model3: adjusted for age, sex, race, education, smoking status, and BMI.

The bold values mean *p* < 0.05.

### MUFAs intake and GC

To further study the associations between MUFAs and risk of GC, the above three models were used to evaluate the odds of GC across the quartiles (Q1: <16.512 g, Q2: 16.512–25.429 g, Q3: 25.429–37.203 g, Q4: >37.203 g) of total intake of MUFAs (Table 4). In all three models, the top quartile of total MUFA intake had over 90% lower likelihood of GC [model1: OR = 0.03, 95% CI: (0.00–0.25), *p* < 0.01; model2: OR = 0.04, 95% CI: (0.00–0.38), *p* = 0.01; model3: OR = 0.04, 95% CI: (0.00–0.40), *p* = 0.01]. These results indicate that a diet rich in MUFAs might play a protective role against GC.

**TABLE 4:** Odds ratios and 95% confidence intervals for GC according to the daily dietary MUFA intake level.

Variable	Model1		Model2		Model3	
	OR (95% CI)	*p*-value	OR (95% CI)	*p*-value	OR (95% CI)	*p*-value
Q1	ref		ref		ref	
Q2	0.9168 (0.1380–6.0891)	0.9277	0.9990 (0.1497–6.6683)	0.9992	1.0181 (0.1508–6.8718)	0.9852
Q3	0.2512 (0.0583–1.0819)	0.0634	0.3106 (0.0727–1.3281)	0.1135	0.3393 (0.0797–1.4449)	0.1419
Q4	0.0260 (0.0028–0.2451)	**0.0017**	0.0400 (0.0043–0.3762)	**0.0053**	0.0426 (0.0045–0.4048)	**0.0065**
P for trend		**0.0023**		**0.0119**		**0.0156**

OR: odds ratio; CI: confidence interval.

Model1: crude model.

Model2: adjusted for age, sex, and race.

Model3: adjusted for age, sex, race, education, smoking status, and BMI.

The bold values mean *p* < 0.05.

The stability of the correlation between MUFAs and GC risk was further confirmed in different populations (Table 5). Analyses stratified by race show that MUFA intake was associated with lower GC risk in black participants [OR = 0.96, 95% CI: (0.92–0.99), *p* = 0.02] and white participants [OR = 0.92, 95% CI: (0.84–1.00), *p* = 0.05]. In stratified analyses based on smoking status, MUFAs were significantly correlated with GC risk in non-smokers [OR = 0.94, 95% CI: (0.89–1.00), *p* = 0.04]. Stratification by BMI showed that MUFAs were significantly associated with lower GC risk only in people with healthy weights [BMI: 18.5–25, OR = 0.89, 95% CI: (0.87–0.91), *p* < 0.0001]. Overall, the findings of this study indicate that a high dietary intake of MUFAs decreases the risk of GC.

**TABLE 5:** Subgroup analysis of the associations between MUFAs and GC.

Variable	OR (95% CI)	*p*-value
Age		
<60	0.9493 (0.8969–1.0049)	0.0726
>60	0.9071 (0.8156–1.0088)	0.0718
Race		
Non-Hispanic Black	0.9572 (0.9220–0.9938)	**0.0227**
Non-Hispanic White	0.9169 (0.8411–0.9995)	**0.0488**
Mexican American	1.0251 (0.9768–1.0758)	0.3103
Other Hispanic	0.9987 (0.9956–1.0019)	0.4225
Other race	0.9823 (0.9059–1.0653)	0.6637
Smoking status		
No	0.9416 (0.8894–0.9969)	**0.0388**
Yes	0.9325 (0.8330–1.0440)	0.2228
BMI		
Underweight	0.8893 (0.8702–0.9088)	**<0.0001**
Healthy weight	0.9733 (0.9350–1.0133)	0.1855
Overweight	0.9304 (0.8538–1.0138)	0.0988
Obese	0.9479 (0.8383–1.0719)	0.3904

The bold values mean *p* < 0.05.

### Causal relationship between MUFAs and GC risk

**FIGURE 2:**
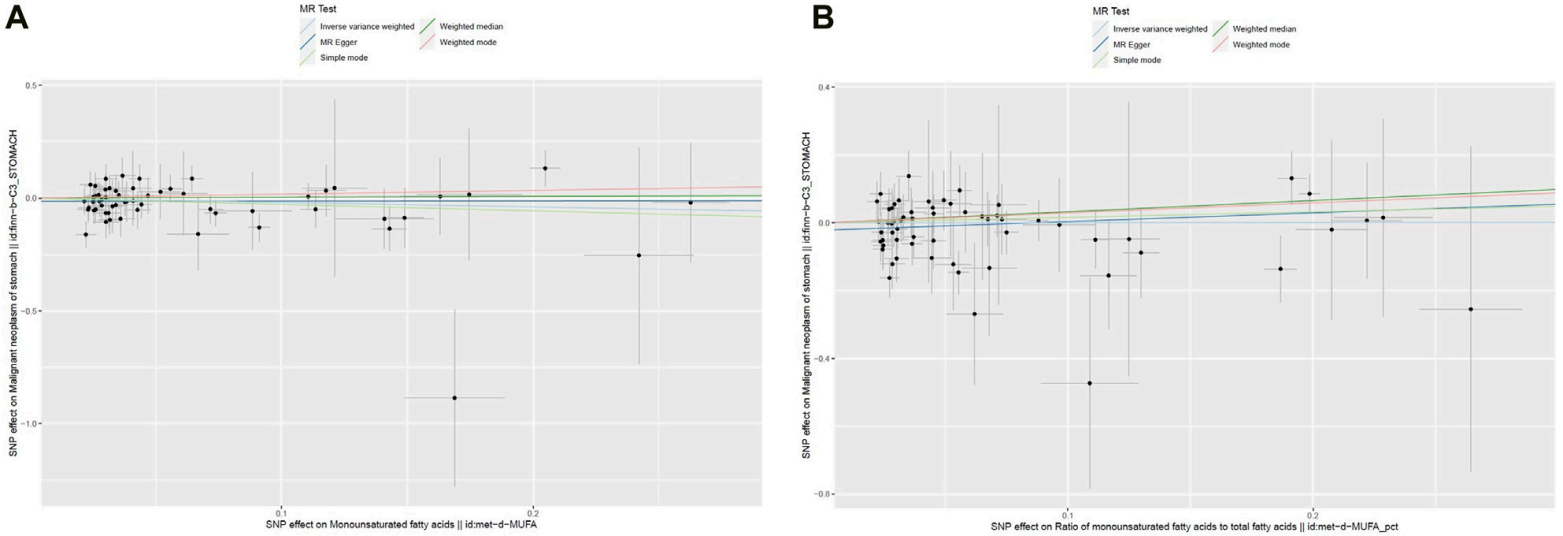
Scatter plots of the genetic associations between **(A)** MUFAs and **(B)** ratio of MUFAs to total fatty acids and GC.

The cross-sectional study design of NHANES prevented the establishment of a causal relationship between the dietary factors and risk of GC. To avoid this limitation, MR analyses were conducted, and details of these SNPs are given in Supplementary Tables S1,S2. The F-statistic for each SNP was above 10. From the results of the IVW analyses, there were no genetic instruments associated with MUFAs or ratio of MUFAs to total fatty acids having a causal relationship with GC risk. The pooled ORs for GC risk in genetically predicted per unit change were 0.82 (95% CI: 0.59–1.14; *p* = 0.23) and 1.00 (95% CI: 0.75–1.35; *p* = 0.98) for MUFAs and ratio of MUFAs to total fatty acids, respectively (Table 6; Figure 2). There was no evidence of heterogeneity or pleiotropy of the aforementioned IVW analysis (Supplementary Table S3).

**TABLE 6:** Causal relationship between MUFAs and GC risk based on different MR methods.

Exposure	MR method	OR	95% CI	*p*-value
MUFAs	IVW	0.8214	0.5939–1.1360	0.2344
	MR–Egger	1.0091	0.5932–1.7165	0.9735
	Weighted-median	1.0375	0.6203–1.7354	0.8883
	Simple mode	0.7523	0.2980–1.8992	0.5493
	Weighted mode	1.1843	0.6340–2.2122	0.5978
Ratio of MUFAs to total fatty acids	IVW	1.0039	0.7475–1.3482	0.9794
	MR–Egger	1.2926	0.8252–2.0246	0.2668
	Weighted-median	1.3905	0.8750–2.2095	0.1630
	Simple mode	1.1772	0.5234–2.6480	0.6946
	Weighted mode	1.3437	0.8680–2.0803	0.1902

OR, odds ratio; CI, confidence interval.

Four other MR methods (MR–Egger, weighted-median, simple mode, and weighted mode) were conducted, and similar results were observed to those of the IVW analyses (Table 6; Figure 2). For both genetic instruments, MR-PRESSO was conducted, and no outliers were found in this study. The leave-one-out sensitivity analysis showed that the overall result could change upon removing rs964184 for both MUFAs and ratio of MUFAs to total fatty acids as well as rs174564 for MUFAs (Supplementary Figure S1).

## Discussion

Numerous studies have unequivocally demonstrated the strong relationships between dietary intake and risk of developing various types of cancers, including lung cancer (Sun et al., 2016), breast cancer (De Cicco et al., 2019), and colorectal cancer (Thanikachalam and Khan, 2019). While some studies have underscored the significant roles of specific nutrients, such as cholesterol (Pih et al., 2021), nitrates (Poorolajal et al., 2020), salt (Wu et al., 2021), and alcohol (Laszkowska et al., 2021), in the incidence and progression of GC, a common tendency in these investigations is the exclusive focus on individual dietary factors that neglects the intricate interplay and complexities of different dietary intakes. It is important to recognize that modifications to a single dietary factor can invariably lead to compensatory changes in other dietary characteristics. Thus, the present cross-sectional study sought to address this deficiency by examining the NHANES database to elucidate the relationships between 32 dietary factors and risk of GC. The findings from a weighted logistic multivariate analysis reveal a noteworthy association between the intake of MUFAs and reduced risk of GC [OR = 0.8343, 95% CI: (0.7572–0.9192), *p* < 0.001]. This underscores the significance of considering the comprehensive dietary landscape for understanding the multiple factors at play in the development of GC.

Extensive research has been conducted on MUFAs owing to their potential health benefits (Snaebjornsson et al., 2020). Foods such as olive oil, avocados, nuts, and seeds, which are common components of the Mediterranean diet, have abundant quantities of MUFAs, and this diet is renowned for its benefits against cardiovascular diseases, obesity, and malignancies (Davis et al., 2015; Schwingshackl et al., 2017; Morze et al., 2021). Current studies advocate the restriction of SFAs and incorporation of higher proportions of MUFAs and PUFAs into a healthy diet (Sacks et al., 2017). Moreover, a recent comprehensive meta-analysis involving 3,202,496 participants revealed an inverse association between the Mediterranean diet and mortality rates of several cancers, including GC, highlighting the potential role of MUFAs in protecting against GC (Morze et al., 2021). Additionally, two separate studies found that dietary MUFAs were linked to a reduced risk of pancreatic cancer (Nkondjock et al., 2005; Banim et al., 2018). The potential antitumor effects of MUFAs may be attributed to their antioxidant properties, capacity to reduce chronic inflammation, and cholesterol-lowering properties (Farag and Gad, 2022; Wan et al., 2022; Guo et al., 2023). Furthermore, in the present NHANES observational study, after adjusting for potential confounders, the authors observed a protective effect of a high-MUFAs diet against GC, consistent with the findings of the aforementioned studies. These findings collectively suggest the potential of MUFAs in mitigating the risk of certain types of cancer while emphasizing their value as a component of a health-promoting diet.

However, some studies have reported conflicting results regarding the association between MUFAs and cancer risk. A large-scale case-controlled study revealed that increased MUFA intake was linked to higher odds of breast cancer (Sasanfar et al., 2022). Similarly, another study reported a positive association between dietary MUFA intake and pancreatic cancer (Gong et al., 2010). Therefore, evidence regarding the relationship between dietary MUFAs and cancer risk remains inconclusive. It is worth noting that there have been no cohort studies to investigate the association between dietary MUFAs and GC to date. This indicates the need for further research to clarify the impacts of MUFA consumption on GC risk.

The primary limitation of an observational study is the challenge of establishing a causal relationship. To address this limitation, two-sample MR analyses were conducted to investigate any potential causal relationships between MUFAs or ratio of MUFAs to total fatty acids and the risk of GC. The initial findings from the IVW analysis do not support a causal relationship between MUFAs or ratio of MUFAs to total fatty acids and GC. Furthermore, this study incorporated four additional MR analyses, all of which consistently aligned with the findings of the IVW analysis, thereby enhancing the robustness of the findings. Despite the seemingly contradictory results between the NHANES observational study and MR analyses, it would be premature to conclusively label MUFAs as “ineffective” in mitigating GC risk. The complexity of dietary factor interactions and metabolism in the human body suggests that compensatory mechanisms may occur when the intake of a specific dietary factor is altered over a short period of time. In light of this intricate interplay, it is more prudent to view each dietary factor as an integral component, akin to individual bricks contributing to the construction of a “great wall” that safeguards against malignancies such as GC.

To the best of the authors’ knowledge, this study is an initial attempt to examine the correlations between dietary MUFAs intake and risk of GC by integrating an observational study with two-sample MR analyses, thereby enhancing the reliability of the findings. Although the results derived from the MR analyses do not substantiate a causal role, it remains imperative to further investigate whether increased consumption of MUFA-rich foods exerts a protective effect against GC.

Despite the findings of this study, several limitations should be considered. First, the relatively lower incidence of GC in the USA compared to East Asia implies a need for further analyses using databases of Asian participants and GWAS to bolster the findings. Second, the use of self-reported 24-h dietary recall data in NHANES may not be fully representative of the participants’ long-term dietary intakes. Third, the absence of information on GC staging, histological findings, surgical history, and fatalities among the study participants precludes subgroup analyses based on the cancer stages, potentially affecting the results. Lastly, this study did not delineate the MUFAs based on their derivation from animal or plant sources, introducing the possibility of biases.

## Conclusion

In conclusion, the results of the present study show no evidence to support a causal link between MUFA intake and gastric cancer risk. Larger studies are therefore required to explore the potential associations between GC risk and animal- or plant-derived MUFAs.

## Data Availability

The original contributions presented in the study are included in the article/Supplementary Material; further inquiries can be directed to the corresponding authors.
