# Supplementary material for: Associations of dietary factors with gastric cancer risk: insights from NHANES 2003–2016 and mendelian randomization analyses
Source: Front Genet. 2024 May 2;15:1377434. doi: 10.3389/fgene.2024.1377434 (PMC11096504; doi:10.3389/fgene.2024.1377434)
Supplement: Supplementary file 3 [file Table4.DOCX]

**Stable3** The heterogeneity or pleiotropy of current MR analysis

| **Exposure** | **MR Methods** | **p-Value for Heterogeneity** | **p-Value for Pleiotropy** |
| --- | --- | --- | --- |
| MUFA | IVW | 0.82 | 0.34 |
|  | MR-Egger | 0.82 |  |
| Ratio of MUFA to total fatty acids | IVW | 0.71 | 0.15 |
|  | MR-Egger | 0.75 |  |
